# Primary Pancreatic Burkitt's Lymphoma: A Case Report and Review of the Literature

**DOI:** 10.1155/2018/5952315

**Published:** 2018-01-18

**Authors:** Venkata Rajesh Konjeti, Gerald M. Hefferman, Sravanthi Paluri, Prerna Ganjoo

**Affiliations:** ^1^Virginia Commonwealth University, Richmond, VA, USA; ^2^Alpert Medical School of Brown University, Providence, RI, USA; ^3^Nephrology Associates, East Providence, RI, USA

## Abstract

Primary pancreatic lymphoma (PPL) is of very rare occurrence as an extra nodal site of Non-Hodgkin's lymphoma (NHL). It represents less than 1% of NHL. Out of which Burkitt lymphoma of pancreas is of a rare presentation. It usually occurs in children and presenting in adults is uncommon. The prevalence of pancreatic Burkitt lymphoma is not known as the incidence is significantly low. Clinical features of PPL are predominantly nonspecific and can become difficult with associated inflammation of pancreas. Differentiation of lymphoma to adenocarcinoma is important as chemotherapy is the main stay of treatment in lymphoma. We report a case of 68-year-old female who presented with nonspecific symptoms and was found to have obstructive jaundice secondary to pancreatic head neoplasm which was proved to be pancreatic Burkitt lymphoma which is a rare presentation.

## 1. Introduction

Non-Hodgkin lymphomas (NHL) are a diverse group of malignancies arising from lymphoid tissue. It is the 8th most common cancer in men and 11th most common cancer in women. It accounts for about 4% of all cancers. The most frequent sites for extra nodal lymphomas, which constitute about 20% to 30% of all lymphomas (peripheral T-cell NHL, 70% to 80%; follicular, 8% to 10%), are the stomach, skin, oral cavity and pharynx, small intestine, and central nervous system (CNS) [1, 2]. Of these primary pancreatic lymphoma is an uncommon extra nodal site of NHL. It accounts for 0.2–2% [3, 4], out of which Burkitt's lymphoma is very rare. There are very few (approximately 20) case reports that are reported in the literature. Most of these are reported in children [5–7]. Identification of Burkitt's lymphoma from others is very crucial as chemotherapy is the mainstay of treatment in pancreatic lymphoma. We report a case of primary pancreatic lymphoma in an adult patient with very low Ca19-9 level.

## 2. Case Report

Patient is a 68-year-old thin female wit PMH of chronic Hepatitis C (acquired through blood transfusion for postpartum hemorrhage), essential hypertension, hypothyroidism, and diverticulosis presented to PCP with belching and abdominal bloating for 4 weeks. She also reported unintentional weight loss of 5 lbs (97 to 92 lbs) over the last few months. Otherwise, complete ROS were negative. Her physical exam was positive for icterus only. Otherwise, the rest of the exam including abdomen was unremarkable without any palpable organomegaly. She was sent to ER for evaluation of abnormal blood work. After getting admitted to the hospital, her lab work showed CHEM 7: Na 134/K 4.1/creatinine 0.87/glucose 66; ALT 133/AST 207/ALP 203/Alb 3.5/total bilirubin 12.1 (direct bilirubin- 8); CBC WBC 11.7/Hb 12.4/platelet 288; CA 19-9 level is 57.

Further workup included the following.

*Abdominal US*. There is heterogeneous mass in right lobe of liver with enlarged adjacent pancreatic head containing 2.8 cm hypoechoic mass.

*CT Abdomen and Pelvis with Contrast*. There is periportal solid 7.5 × 4.9 × 12.7 cm mass which obstructs the central hepatic ducts and encases the main portal vein; it is difficult to separate the lesion from liver and pancreatic parenchyma.

*ERCP*. This is a limited study as the duct could not be cannulated. A small duodenal mucosal tear was seen and managed with clips. 

*EGD*. There is firm gastric extrinsic compression in stomach body.

Subsequently a 10 Fr transpapillary biliary drain was placed by IR on 5/9→ findings of long smooth stenosis from right and left hepatic ducts to the papilla. Biliary ductal brushings were sent for cytology.

Patient was started on Zosyn and finished 7-day treatment course. Her hospital course was complicated by continued leakage around the drain site and unsuccessful drainage which lead to internalization of biliary stents. During that time biopsy of the pancreatic head was also obtained. CBD brushing cytology showed atypical lymphocytes suspicious for malignant lymphoma. FNA of pancreatic head showed Burkitt's lymphoma. Pancreatic head biopsy resulted in high grade B-cell lymphoma consistent with Burkitt's lymphoma. The histological/immunohistochemical and cytogenetic features of the mass are described as follows.

### 2.1. Histological Features

The pancreatic head mass needle biopsy morphologically showed sheets of medium-to-large size lymphoid cells with scant-to-moderate amounts of azurophilic cytoplasm, round-to-irregular nuclei with fine basophilic chromatin. Macrophages containing karyorrhectic debris are identified in the background. Immunohistochemically, the lesional cells are positive for CD20, CD10, and bcl-6. A subset shows rare c-myc positivity (control is also weak). They are negative for CD3, TdT, and bcl-2. Ki-67 displays a proliferation index >95%. These morphologic and immunohistochemical features are diagnostic for a high grade B-cell lymphoma and most consistent with Burkitt's lymphoma.

### 2.2. Immunohistochemistry

See Table 1.

### 2.3. Cytogenetic Results

(1) FISH showed no fusion of regions MYC and IGH of chromosomes 8 and 14, respectively.

(2) FISH showed no fusion of IGH and BCL2 regions of chromosomes 14 and 18, respectively.

(3) FISH revealed a splitting of the signal for MYC (8q24.1) in 91% of interface cells examined. This is indicative of MYC rearrangement, most likely a translocation.

(4) FISH did not reveal splitting or other rearrangements of 3q27 (BCL6).

Subsequently oncology team was involved and patient had a bone marrow biopsy done which showed B-cell lymphoma. PET scan showed peritoneal carcinomatosis and malignant ascites. Patient was started on chemotherapy regimen consisting of etoposide, prednisone, vincristine (Oncovin), and doxorubicin hydrochloride (hydroxydaunorubicin hydrochloride). After her first dose of chemotherapy she became encephalopathic and MRI of brain at that time showed possible involvement of base of skull and she was started on intrathecal methotrexate. Lumbar puncture was done subsequently and the CSF analysis was negative for infection and malignancy.

She received total of two cycles of chemotherapy and was admitted to hospital with sepsis and bacteremia. She deteriorated very quickly during that hospitalization and family opted for hospice and patient passed away in the hospice.

## 3. Discussion

The increasing use and sensitivity of cross-sectional imaging in identifying pancreatic lesions has led to a corresponding increase in the detection pancreatic tumors. While pancreatic ducal adenocarcinoma represents the majority of such cases, other, less common pancreatic neoplasms comprise an important fraction of pancreatic cancers. Primary pancreatic lymphoma (PPL) represents a significant class within these less common diagnoses [8]. Patients with either neoplasm may present with similar clinical manifestations; however, as the effective treatments for adenocarcinomas and lymphomas differ greatly, it is critical that an accurate diagnosis be made [9].

PPLs are extremely rare, comprising less than 2% of extra nodal malignant lymphomas and 0.5% of pancreatic tumors [3, 4], although nearly one-third of non-Hodgkin's lymphoma patients will ultimately develop some form of pancreatic involvement [10]. Diffuse large-cell lymphomas comprise the bulk of PPLs [8]. A less common form, Burkitt's lymphoma, is exceptionally rare, with fewer than 20 reported cases worldwide as of 2013 [11].

There are three subtypes of Burkitt's lymphoma: endemic, sporadic, and immunodeficiency- associated. The endemic form, described by Irish surgeon Denis Burkitt, is endemic to Papua New Guiney and Sub-Saharan Africa [12], mainly involves the mandible and maxilla, and is uniformly associated with Epstein-Barr virus (EBV) infection. The sporadic form is rare, with an incidence of 0.2–0.3 per 100,000 people per year globally, accounting for 30% of pediatric lymphomas and less than 1% of adult non-Hodgkin's lymphomas in the United States and Europe. These sporadic- form neoplasms are EBV associated at a rate of only 10–20% [13]. The immunodeficiency-associated variant of the disease, a common neoplasm of HIV infected patients, is generally EBV-negative outside of Africa and strongly associated with EBV within the continent [14].

Histologically, Burkitt's lymphoma presents with monomorphic, medium-sized B-cells with many mitotic figures and basophilic cytoplasm. High rates of apoptosis with benign histiocytic phagocytosis of apoptotic debris may lead to a starry-sky growth pattern [15]. The neoplastic B-cells of Burkitt's lymphoma have several clinically useful distinguishing immunophenotypic features. They are positive for the following: IgM surface immunoglobulin, surface light chains, CD10, CD19, CD20, CD22, CD43, CD79a, HLA-DR, and BCL-6. Additionally, Epstein-Barr virus-associated forms will express the EBV/C3d receptor, CD21. The neoplasms are negative for the following: CD5, CD23, BCL-2, and TdT [13]. Ki-67 expression in these tumors, a proxy measurement for growth fraction [11], approaches 100% [13].

Dysregulation of the MYC gene is a defining molecular marker of Burkitt's lymphoma. This is primarily a consequence of translocations between the MYC gene on chromosome 8 and the IgH gene on chromosome 14 [t(8;14)(q24;q32)], although MYC-*κ* light chain and MYC-*λ* light chain translocations [t(2;8)(p12;q24) and t(8;22)(q24;q11), resp.] are also described [13, 16]. Importantly, the absence of a MYC translocation does not rule out a diagnosis of Burkitt's lymphoma; in up to 10% of cases, fluorescence in situ hybridization is negative for these translocations. In these instances, MYC activation may be the result of miRNA deregulation or other causes [17].

Clinical symptoms of PPL are often non-specific [9] and can be made more difficult by inflammation of the pancreas [18]. As a consequence, clinical presentation may be of little help in distinguishing Burkitt's lymphoma from other pancreatic tumors [4, 19]. However, since treatments for pancreatic adenocarcinoma and other lymphomas of the pancreas differ significantly, differentiation of Burkitt's lymphoma from other pancreatic neoplasms is crucial [4, 9, 18]. Diagnosis can typically be made following nonsurgical biopsy of the tumor lesion [4, 11, 20].

Unlike pancreatic adenocarcinoma, which is treated surgically, chemotherapy has been shown to be the most efficacious first-line treatment for PPL [3, 8]. Common chemotherapeutic regimens directed against PPL include CVP (cyclophosphamide, vincristine, and prednisone), MACOP-B (methotrexate, adriamycin, cyclophosphamide, vincristine, prednisone, and bleomycin), and CHOP (cyclophosphamide, doxorubicin, vincristine, and prednisone). The further addition of rituximab, a monoclonal antibody targeting the CD20 B-cell antigen, to the CHOP regimen (R-CHOP) has been shown to enhance complete response rate, event-free survival, and overall survival in patients with diffuse large B-cell lymphoma. The role of radiation therapy in PPL remains poorly defined [3].

Multiple intensive chemotherapeutic approaches have demonstrated excellent efficacy in the treatment of Burkitt's lymphoma more generally; however, due to the relative lack of randomized trials directed towards the treatment of the disease, an optimal initial therapeutic approach has not been clearly defined [13]. This limitation is further compounded in the primary pancreatic form of the disease by its relative rarity. However, present chemotherapeutic approaches, comprising doxorubicin, alkylators, vincristine, and etoposide in combination with directed therapy targeting the prevention and eradication of CNS disease, have shown excellent outcomes. These treatments, described in detail by Jacobson and LaCasce, include CODOX-M/IVAC, the cancer and leukemia group-B regimen, hyperCVAD with and without rituximab, the lymphoma malign B regimen, and DA-REPOCH [13].

To conclude, PPLs are a class of rare cancers affecting the pancreas, of which Burkitt's lymphoma comprises an exceptionally rare subset. However, as efficacious treatments for the disease exist and differ markedly from that of pancreatic adenocarcinoma, it is important that PPLs more generally and Burkitt's lymphoma in particular be included in the differential when evaluating a probable pancreatic neoplasm.

## Figures and Tables

**Table 1:** 

Antibody	Result
CD10 (M; 56C6; DAKO)	Positive in lesional cells
CD20 (M; L26; Vector)	Positive in lesional cells
CD3 (P; DAKO)	Negative in lesional cells
Bcl-2 (M; 124; DAKO)	Negative in lesional cells
Bcl-6 (M; PG-B6p; DAKO)	Positive in lesional cells
C-myc (RM; Y69; Epitomics)	Focal weak positivity (control is also weak)
Ki- 67 (M; MIB1; DAKO)	>90% positive
TdT (terminal deoxynucleotidyl transferase) (M; SEN28; Vector)	Negative
